# An Acquisition Method for Visible and Near Infrared Images from Single CMYG Color Filter Array-Based Sensor

**DOI:** 10.3390/s20195578

**Published:** 2020-09-29

**Authors:** Younghyeon Park, Byeungwoo Jeon

**Affiliations:** Department of Electrical and Computer Engineering, Sungkyunkwan University, Suwon 16419, Korea; neversky@skku.edu

**Keywords:** cameras, image processing, infrared imaging, near infrared, photography

## Abstract

Near-infrared (NIR) images are very useful in many image processing applications, including banknote recognition, vein detection, and surveillance, to name a few. To acquire the NIR image together with visible range signals, an imaging device should be able to simultaneously capture NIR and visible range images. An implementation of such a system having separate sensors for NIR and visible light has practical shortcomings due to its size and hardware cost. To overcome this, a single sensor-based acquisition method is investigated in this paper. The proposed imaging system is equipped with a conventional color filter array of cyan, magenta, yellow, and green, and achieves signal separation by applying a proposed separation matrix which is derived by mathematical modeling of the signal acquisition structure. The elements of the separation matrix are calculated through color space conversion and experimental data. Subsequently, an additional denoising process is implemented to enhance the quality of the separated images. Experimental results show that the proposed method successfully separates the acquired mixed image of visible and near-infrared signals into individual red, green, and blue (RGB) and NIR images. The separation performance of the proposed method is compared to that of related work in terms of the average peak-signal-to-noise-ratio (PSNR) and color distance. The proposed method attains average PSNR value of 37.04 and 33.29 dB, respectively for the separated RGB and NIR images, which is respectively 6.72 and 2.55 dB higher than the work used for comparison.

## 1. Introduction

The widespread use of cameras in daily life has spurred numerous applications. These applications can become even more powerful if they can leverage more useful imaging information at lower cost. Active investigations have been underway to extract unconventional information from the images taken by inexpensive cameras for usage beyond the simple viewing of a photograph. For example, capturing images at non-visible wavelengths by inexpensive cameras could prove incredibly useful. One such effort of immediate interest is the acquisition of signals ranging 780–1400 nm of near-infrared (NIR) wavelengths [1].

Conventional consumer-level cameras only acquire images in the visible wavelength region, typically by using a color filter array (CFA) placed in front of the image sensor. To avoid saturation from the accompanying infrared (IR) signal, an IR cut filter is also placed in front of the image sensor [2]. This configuration acquires an image that covers the wavelength range 380–780 nm [1] that the human eye normally sees. However, signals in the infrared range can provide valuable additional information, as indicated by numerous investigations that have been conducted on how to best extract more useful visual information from the infrared signal [3].

NIR has different spectral characteristics than visible (VIS) wavelengths [2]. Many industrial applications have applied NIR imaging to enhance the quality of images in the visible range. In remote sensing, NIR images are quite effective in identifying forests since vegetation has high reflectivity in the NIR [4]. High NIR penetration into water vapor also explains why NIR images give clear images even in hazy weather conditions, and this characteristic has been successfully exploited to dehaze outdoor scenes [5].

In manufacturing applications, NIR can detect defective products or bruises on fruit [6,7,8]. In medical imaging, NIR helps to detect veins, which is quite useful for intravenous injections in the arm [9,10]. In surveillance imaging, NIR light sources have been used for night vision imaging without disturbing subjects being imaged. NIR images can also be utilized to reduce noise in visible range images captured in low light conditions [11,12]. Even this small sampling of NIR applications clearly demonstrates that NIR imaging is extremely important. These applications can be further enhanced by the combination of VIS and NIR images. In capturing both VIS and NIR images from a scene, simultaneous capture of both of them by a single system can make matters extremely simple by avoiding necessity of registration.

The conventional 2-CCD RGB-IR camera [13] has been used to simultaneously capture both visible red, green, and blue (RGB), and IR images. Its drawbacks include limited portability and high cost, as the system consists of two sensors and a beam splitter. The two sensors are placed 90 degrees apart to capture the scene at the same position. This dual sensor-based system incurs double hardware production costs and is bigger than a single sensor-based system. To overcome these limitations, research has been devoted to obtain both visible and IR images with a single sensor. For instance, the transverse field detector (TFD)-based VIS and NIR image acquisition method operates under the principle of different spectral transmittance in silicon [14]. However, the TFD-based imaging system is still in its experimental phase. Another notable approach is the novel design of a CFA. For instance, Koyama et al. [15] proposed a photonic crystal color filter (PCCF)-based approach which adds a defect layer in a TiO_2_ and SiO_2_ stacked photonic crystal. The thickness of the defect layer controls the band-gap length between the visible and infrared wavelengths. The detector can be designed with an array of four different defect layer thicknesses to obtain R+IR, G+IR, B+IR, and IR bands. Lu et al. [16] proposed an optimal CFA design which simulates to get an optimal CFA pattern with a various number of color filters. Because of the high production cost of PCCFs and Lu’s CFA, it is difficult to utilize them in low-cost consumer devices. Although a more pragmatic camera module has been developed using an RGB-IR CFA [17,18,19,20], it replaces one pixel of green band pass filter in the CFA pattern with an IR long pass filter and its production cost is higher than visible range color filters. Sadegnipoor et al. took a different approach that used a single RGB camera, employing a compressive sensing framework and developed a signal separation method [21] to extract the VIS and NIR images from a mixed image signal. The hardware system can be easily implemented by just removing the IR cut filter from a conventional camera. However, the proposed separation method has problems with image quality and heavy computational complexity due to the ill-posed mathematical structure and consequential iterative reconstruction process for compressively sensed data.

Our prior work [22] took a similar approach but with the complementary CFA of cyan, magenta, yellow, and green (CMYG) instead of the RGB primary colors. CMYG CFAs have the benefit of better sensitivity than RGB CFAs because of the wider spectrum range of each color filter [23,24]. However, the mathematical model in the prior work considered only the noise-free case. Nontrivial noise was observed in the experimentally separated images [22].

In this paper, we improve on our prior work by re-formulating the starting mathematical model. In the proposed mathematical model, the color space conversion is more rigorously performed by converting from CMYG color space to the device independent color space of the standard CIE1931 XYZ [25]. Moreover, by adding an additive noisy term to the proposed mathematical model, the noise characteristic is analyzed, and subsequently, color-guided filtering [26] is applied to minimize the noise after the proposed separation process.

**Figure 1:**
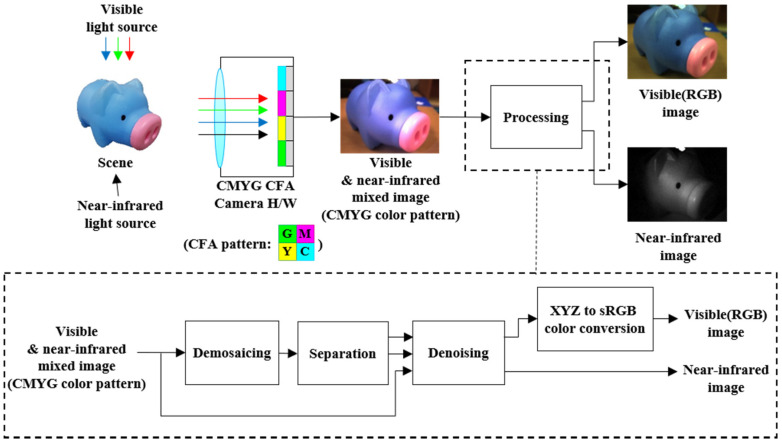
The proposed visible and near-infrared imaging system structure.

Figure 1 depicts the structure of the proposed method. The proposed imaging system is made by removing the IR cut filter from a conventional camera. The image is taken under several lighting conditions which contain visible and NIR wavelengths. The proposed imaging system captures a monochrome mosaic pattern image and each pixel intensity corresponds to a color filter of the CMYG CFA pattern. To generate a color image from the monochrome image, a demosaicing process, such as bilinear interpolation [27] method, is applied. After the demosaicing process, a four-color channel of the mixed input image is generated, which contains mixed signals from the visible and NIR spectrums. In the separation process, the mixed input image is separated into VIS and NIR images. In the denoising process, the noise in the separated XYZ and NIR images is minimized by the color-guided filtering method [26] which utilizes the four-channel input image as the guide image. The separated visible image in XYZ color space [25] can be changed to the color space of choice, such as the standard RGB (sRGB) [28]. The proposed separation, denoising, and color conversion processes generate both visible and NIR images using a conventional single imaging sensor. We note that the proposed method allows simultaneous acquisition of both images and does not require an additional registration process to match different temporal and spatial viewpoints.

The remainder of this paper is organized as follows. Section 2 addresses the mathematical model and the proposed separation method. Experiment results are given in Section 3, and the conclusion is provided in Section 4.

## 2. Proposed Method

### 2.1. Mathematical Model

The image capturing mechanism used in this paper is exactly the same as conventional camera systems. The light rays reflected from a point on the object’s surface goes through the lens. After the light rays pass through the lens, an IR cut filter reflects the NIR wavelengths away while only passing visible wavelengths of light in. The installed CFA in front of the image sensor separates the incoming wavelengths into the appropriate color channels. The number of channels and colors used in the CFA depends on the system characteristics. The light rays passing through the optical lens and filters are integrated by the image sensor to form a corresponding pixel value. The mathematical model for each pixel value of the conventional camera system can be represented as

(1) $$d_{F}^{conv.}(k)=\int_{\lambda_{VIS}}T_{F}(\lambda ,k)E(\lambda ,k)R(\lambda ,k)d\lambda +e_{F}(k)$$

where *F* is a specific color channel, *k* indicates the spatial location of a pixel, $d_{F}^{conv.}\left(k\right)$ is the intensity at the *k*^th^ pixel position sensed by a sensor of a color channel *F*, $T_{F}\left(\lambda ,\;k\right)$ is the spectral distribution of transmittance by the color filter array for *F*, E(λ,k) is the spectral distribution of the light source, R(λ,k) is the spectral distribution of object reflectance in the scene, $e_{F}\left(k\right)$ is the temporal dark noise [29] characteristic of the sensor, and $\lambda_{VIS}$ refers to range of visible wavelength.

A conventional camera system captures the visible range image only with the help of the IR cut filter, and concurrent capture of the NIR image is possible by removing the IR cut filter. When the IR cut filter is removed, (1) can be changed to

(2) $$d_{F}(k)=\int_{\lambda_{VIS}}T_{F}(\lambda ,k)E(\lambda ,k)R(\lambda ,k)d\lambda \;+\int_{\lambda_{NIR}}T_{F}(\lambda ,k)E(\lambda ,k)R(\lambda ,k)d\lambda +e_{F}(k)$$

where $d_{F}\left(k\right)$ represents the intensity at the *k*^th^ pixel position sensed by a sensor of a color channel *F*, and $\lambda_{NIR}$ refers to range of NIR wavelength, and the intensity includes both visible and NIR wavelengths. In this paper, the four-color channels of F∈ {C, M, Y, G} are employed with a CMYG CFA. The intensity $d_{F}(k)$ sensed by a sensor of a color channel *F* according to the light integration model of (2) can be written as

(3) $$d_{F}(k)=d_{F}^{VIS}(k)+d_{F}^{NIR}(k)+e_{F}(k)$$

where $d_{F}^{VIS}\left(k\right)$ and $d_{F}^{NIR}\left(k\right)$ are the pixel intensities due to visible and NIR wavelengths, respectively. In (3), $d_{F}^{NIR}(k)$ is the NIR intensity sensed by a sensor of each color channel; that is, $d_{C}^{NIR}(k)$, $d_{M}^{NIR}(k)$, $d_{Y}^{NIR}(k)$, and $d_{G}^{NIR}(k)$. The NIR intensity sensed in each color channel, $d_{F}^{NIR}(k)$, is modeled as $d_{F}^{NIR}(k)=\omega_{F}d^{NIR}(k)$ where *ω_F_* (that is, *ω_C_*, *ω_M_*, *ω_Y_*, and *ω_G_*) is a weighting factor of the NIR transmittance on each channel of the CFA, and $d^{NIR}(k)$ is the NIR intensity unaffected by the CFA. Using this representation, (3) can be reformulated as

(4) $$d_{F}(k)=d_{F}^{VIS}(k)+\omega_{F}d^{NIR}(k)+e_{F}(k)$$

More detailed information about the linear relation in (4) will be discussed later along with experimental verification. Equation (4) can be represented in the following matrix form:

(5) $$\left[\begin{array}{c} d_{C}^{VIS}(k)+\omega_{C}d^{NIR}(k)\\ d_{M}^{VIS}(k)+\omega_{M}d^{NIR}(k)\\ d_{Y}^{VIS}(k)+\omega_{Y}d^{NIR}(k)\\ d_{G}^{VIS}(k)+\omega_{G}d^{NIR}(k) \end{array}\right]=\left[\begin{array}{ccccc} 1 & 0 & 0 & 0 & \omega_{C}\\ 0 & 1 & 0 & 0 & \omega_{M}\\ 0 & 0 & 1 & 0 & \omega_{Y}\\ 0 & 0 & 0 & 1 & \omega_{G} \end{array}\right]\left[\begin{array}{c} d_{C}^{VIS}(k)\\ d_{M}^{VIS}(k)\\ d_{Y}^{VIS}(k)\\ d_{G}^{VIS}(k)\\ d^{NIR}(k) \end{array}\right]$$

The matrix form in (5) represents a linear relationship between visible and NIR mixed input and the separated output. The separated four channel CMYG color-based visible output values depend on the device characteristics. Therefore, the CMYG color space is not appropriate to obtain consistent colors on different devices. To produce device independent color in this paper, CMYG color space is converted into the standard CIE1931 XYZ color space [25] with

(6) $$\left[\begin{array}{c} d_{C}^{VIS}(k)\\ d_{M}^{VIS}(k)\\ d_{Y}^{VIS}(k)\\ d_{G}^{VIS}(k) \end{array}\right]=\left[\begin{array}{ccc} \alpha_{11} & \alpha_{12} & \alpha_{13}\\ \alpha_{21} & \alpha_{22} & \alpha_{23}\\ \alpha_{31} & \alpha_{32} & \alpha_{33}\\ \alpha_{41} & \alpha_{42} & \alpha_{43} \end{array}\right]\left[\begin{array}{c} d_{X}^{VIS}(k)\\ d_{Y}^{VIS}(k)\\ d_{Z}^{VIS}(k) \end{array}\right]$$

where the $\alpha_{ij}$’s are the color conversion coefficients, and $d_{X}^{VIS}(k)$, $d_{Y}^{VIS}(k)$, and $d_{Z}^{VIS}(k)$ are the intensities of the visible image in XYZ color space. Supplementing (6) with the NIR component gives

(7) $$\left[\begin{array}{c} d_{C}^{VIS}(k)\\ d_{M}^{VIS}(k)\\ d_{Y}^{VIS}(k)\\ d_{G}^{VIS}(k)\\ d^{NIR}(k) \end{array}\right]=\left[\begin{array}{cccc} \alpha_{11} & \alpha_{12} & \alpha_{13} & 0\\ \alpha_{21} & \alpha_{22} & \alpha_{23} & 0\\ \alpha_{31} & \alpha_{32} & \alpha_{33} & 0\\ \alpha_{41} & \alpha_{42} & \alpha_{43} & 0\\ 0 & 0 & 0 & 1 \end{array}\right]\left[\begin{array}{c} d_{X}^{VIS}(k)\\ d_{Y}^{VIS}(k)\\ d_{Z}^{VIS}(k)\\ d^{NIR}(k) \end{array}\right]$$

By substituting (7) into (5), the following equation can be found:

(8) $$\left[\begin{array}{c} d_{C}^{VIS}(k)+\omega_{C}d^{NIR}(k)\\ d_{M}^{VIS}(k)+\omega_{M}d^{NIR}(k)\\ d_{Y}^{VIS}(k)+\omega_{Y}d^{NIR}(k)\\ d_{G}^{VIS}(k)+\omega_{G}d^{NIR}(k) \end{array}\right]=C\left[\begin{array}{c} d_{X}^{VIS}(k)\\ d_{Y}^{VIS}(k)\\ d_{Z}^{VIS}(k)\\ d^{NIR}(k) \end{array}\right]\;\mathrm{where}\;C=\left[\begin{array}{cccc} \alpha_{11} & \alpha_{12} & \alpha_{13} & \omega_{C}\\ \alpha_{21} & \alpha_{22} & \alpha_{23} & \omega_{M}\\ \alpha_{31} & \alpha_{32} & \alpha_{33} & \omega_{Y}\\ \alpha_{41} & \alpha_{42} & \alpha_{43} & \omega_{G} \end{array}\right]$$

In (8), **C** is called the combination matrix. By applying (8), (3) can be rewritten as

(9) $$\left[\begin{array}{c} d_{C}(k)\\ d_{M}(k)\\ d_{Y}(k)\\ d_{G}(k) \end{array}\right]=C\left[\begin{array}{c} d_{X}^{VIS}(k)\\ d_{Y}^{VIS}(k)\\ d_{Z}^{VIS}(k)\\ d^{NIR}(k) \end{array}\right]+\left[\begin{array}{c} e_{C}(k)\\ e_{M}(k)\\ e_{Y}(k)\\ e_{G}(k) \end{array}\right]$$

In this paper, the separation matrix, **S**, is defined as

(10) $$S=C^{-1}$$

Finally, the separated XYZ and NIR pixel intensities at *k^th^* position can be found with

(11) $$S\left[\begin{array}{c} d_{C}(k)\\ d_{M}(k)\\ d_{Y}(k)\\ d_{G}(k) \end{array}\right]=\left[\begin{array}{c} d_{X}^{VIS}(k)\\ d_{Y}^{VIS}(k)\\ d_{Z}^{VIS}(k)\\ d^{NIR}(k) \end{array}\right]+S\left[\begin{array}{c} e_{C}(k)\\ e_{M}(k)\\ e_{Y}(k)\\ e_{G}(k) \end{array}\right]=\left[\begin{array}{c} {\hat{d}}_{X}^{VIS}(k)\\ {\hat{d}}_{Y}^{VIS}(k)\\ {\hat{d}}_{Z}^{VIS}(k)\\ {\hat{d}}^{NIR}(k) \end{array}\right]$$

where ${\hat{d}}_{X}^{VIS}(k)$, ${\hat{d}}_{Y}^{VIS}(k)$, ${\hat{d}}_{Z}^{VIS}(k)$, and ${\hat{d}}^{NIR}(k)$ are the separated visible (in XYZ color space) and NIR pixels containing temporal dark noise. The noise analysis and corresponding denoising process will be discussed later.

### 2.2. Color Conversion to XYZ Color Space

In this paper, the CMYG CFA is used to capture the color in the visible wavelengths. The input image has four color channels. On the other hand, the display system controls the color in three channels of RGB color space so that the input image in CMYG color space should be changed into the RGB color space. The conventional way to do this conversion is to change the input intensity values of the sensor to the target standard color space. In this paper, we convert CMYG input to the standard CIE1931 XYZ [25] color space.

From (6), the relation between CMYG and XYZ is linear. The color conversion can be done by linear regression [28] by excluding the bias term, making it easy to adapt to the proposed model. In the process of training the color conversion coefficients by linear regression, the selection of the color temperature of the light source and the reference target color chart are important. The color temperature of a light source can be affected by the white balance of the image. If the color was trained under a 6500K fluorescent light, the white balance is only matched under the same light source. In case of the reference target color, the standard Macbeth color chart [30] is widely used to train the color space conversion coefficients in (6).

After finding the color conversion coefficients from CMYG to XYZ space, the converted XYZ space can be converted to RGB color space for display on a device. In this paper, we apply the conversion to sRGB [31] which is the predominantly used color space for display of photos in the internet [32].

### 2.3. Calculation of the NIR Weighting Coefficient

Section 2.2 explained how to derive the color space conversion coefficients in (8). This section describes how to find the value of the NIR weighting coefficients *ω_C_, ω_M_*, *ω_Y_*, and *ω_G_* in (8). The proposed method estimates the weighting coefficients by finding the relative ratio between the coefficients. To find the relative ratio, a weighting coefficient of a specific channel is treated as a reference value. In this paper, we set the NIR intensity in the green channel of input image as the reference. To calculate the coefficients, (4) is slightly rewritten as

(12) $$\omega_{F}d^{NIR}(k)=\frac{\omega_{F}}{\omega_{G}}\omega_{G}d^{NIR}(k)=\omega_{FG}\omega_{G}d^{NIR}(k)$$

where $\omega_{FG}$ is the ratio of $\omega_{F}$ against $\omega_{G}$, that is, $\omega_{F}/\omega_{G}$, *F* ∈ {*C*, *M*, *Y*, *G*}, and $\omega_{GG}$ = 1. By reflecting (8), the combination matrix (**C**) also can be changed to

(13) $$C=\left[\begin{array}{cccc} \alpha_{11} & \alpha_{12} & \alpha_{13} & \omega_{CG}\omega_{G}\\ \alpha_{21} & \alpha_{22} & \alpha_{23} & \omega_{MG}\omega_{G}\\ \alpha_{31} & \alpha_{32} & \alpha_{33} & \omega_{YG}\omega_{G}\\ \alpha_{41} & \alpha_{42} & \alpha_{43} & \omega_{G} \end{array}\right]$$

To find the values of *ω_CG_*, *ω_MG_*, and *ω_YG_*, an experiment was performed. To capture an image which contains only the NIR information, the camera captures a scene which is illuminated with an NIR light source only. The image was taken with an 850 nm NIR light source, so the image only contains reflected NIR light from a white surface. The light source of visible wavelength is absent. By the experiment, (4) by reflecting (13) can be expressed as

(14) $$d_{F}(k)=\omega_{FG}\omega_{G}d^{NIR}(k)+e_{F}(k)$$

From (14), it can be estimated that only reflected NIR light is projected onto the sensor after passing through the CMYG CFA. Therefore, the value of the weighting factor only depends on the sensor characteristic and the transmittance of the CMYG CFA.

**Figure 2:**
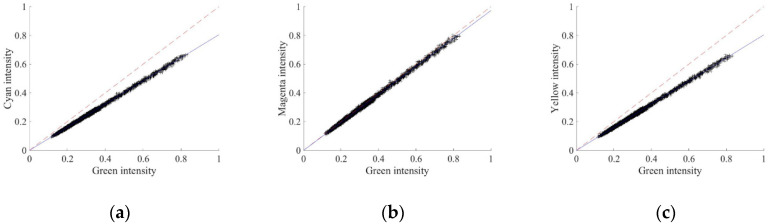
The Near-infrared (NIR) intensity scatter plot between two different color channels (**a**) Green-Cyan, (**b**) Green-Magenta, and (**c**) Green-Yellow.

Figure 2 depicts scatter plots of NIR intensity between two color channels. It clearly shows a linear relationship between the color channels and the slopes of the linear regressions to the scatter plots imply the values of *ω_CG_*, *ω_MG_*, and *ω_YG_*. Their values are computed by setting the value of *ω_G_* as 1, which implies that the amount of NIR light passing through the green color filter is treated as a relative reference value of the output NIR image.

### 2.4. Noise Analysis

**Figure 3:**
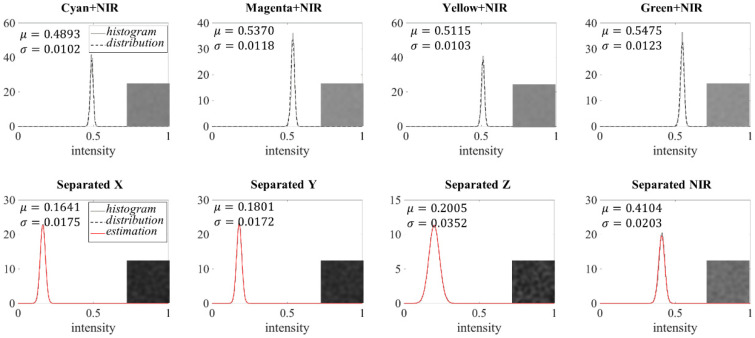
The noise distribution of the input and the separated images (in images of grey surface object taken under fluorescent and 850 nm light).

After the separation by multiplying the separation matrix (**S**) and input image pixels, noise are noticeable in the separated visible and NIR images due to the following noise characteristic that is defined as the separation noise in this paper. Equation (11) contains the noise model after the separation process. The noise in each separated image channel is represented as the weighted sum of the input temporal dark noise and each element in the separation matrix. It implies that the separation noise depends on the input temporal dark noise and the separation matrix. Experimental data can elucidate the relationship of noise in the input and the separated images. Figure 3 shows a histogram of the grey reference target taken with the proposed imaging system under fluorescent and 850 nm NIR light. The variance of the captured grey reference target image can be treated as that of the temporal dark noise. In this paper, we assume the noise Gaussian, and the distribution is estimated from the shape of the histogram in Figure 3. To check the relationship between the input noise and the separation noise, following weighted sum of Gaussian random variables [33] is calculated. In general, if

(15) $$e_{F}\sim N(\mu_{F},\sigma_{F}^{2}),F\in \{C,M,Y,G\}$$

then,

(16) $$e_{\left\{X,Y,Z,NIR\right\}}=\sum_{F\in \{C,M,Y,G\}}s_{F}e_{F}\;\sim N\left(\sum_{F\in \{C,M,Y,G\}}s_{F}\mu_{F},\sum_{F\in \{C,M,Y,G\}}{\left(s_{F}\sigma_{F}\right)}^{2}+2\sum_{F_{i},F_{j}\in \{C,M,Y,G\}}s_{F_{i}}s_{F_{j}}Cov(e_{F_{i}},e_{F_{j}})\right)$$

where $\mu_{F}$ and $\sigma_{F}^{2}$ are the mean and variance of the temporal dark noise distribution of input image and *N* indicates the Gaussian probability density function. *S_F_* represents each element in the separation matrix and *e_F_* is the temporal dark noise level on the corresponding channel. The “estimation” in Figure 3 shows the estimated noise distribution of the separated image using (16) with the input noise distribution. The estimated result exactly matches with the histogram of the separated image. From this result, it is expected that the noise in the separated images are fully derived from the temporal dark noise of the input image.

### 2.5. Denoising Method

In Figure 3, it is observed that the variance of noise is larger in the separated images than in the input image. To reduce the noise on the separated images, an effective denoising technique should be applied. In general, we can apply the denoising techniques on the separated images directly. The BM3D filtering [34] is a good example of denoising that can be potentially considered in this case. In Section 2.4, we discovered that the noise distribution in the separated image is formed by the weighted sum of the noise in each input image channel. This implies that if the denoising is applied to the input mixed image, the noises in separated images will be also reduced. However, it is hard to estimate the amount of noise in the input image. In a different approach, the input image can be used as a guidance image to reduce noise of the separated images. If the output image has a linear relationship with a guidance image, the noise can be minimized by deriving the low noise of the guidance image. A denoising technique called color-guided filtering [26] exploits the linear relationship between the noisy image and the guide image. If there is a linear relationship between the input and the separated results, the proposed method is well matched to the guided filtering approach. The color-guided filtering method [26] is applied in this paper to minimize the separation noise by applying the input CMYG image as a guide image. According to [26], a color-guided filter with CMYG can be applied by,

(17) $${\tilde{d}}_{X}^{VIS}(k)=a_{X}^{Τ}(k)\left[\begin{array}{c} d_{C}(k)\\ d_{M}(k)\\ d_{Y}(k)\\ d_{G}(k) \end{array}\right]+b_{X}(k)$$

where ${\tilde{d}}_{X}^{VIS}(k)$ is the *k*^th^ pixel of the filtered separated image of X channel. $a_{X}^{Τ}(k)$ and $b_{X}(k)$ can be calculated by,

(18) $$a_{X}(k)={\left(\Sigma_{k}+\varepsilon U\right)}^{-1}\left(\frac{1}{\left|w\right|}\sum_{i\in w_{k}}\left[\begin{array}{c} d_{C}(i)\\ d_{M}(i)\\ d_{Y}(i)\\ d_{G}(i) \end{array}\right]\;{\hat{d}}_{X}^{VIS}(i)-\mu_{k}{\bar{\hat{d}}}_{X}^{VIS}(k)\right)$$

(19) $$b_{k}={\hat{d}}_{X}^{VIS}(k)-a_{X}^{Τ}(k)\mu_{k}$$

where $a_{X}(k)$ is a 4 × 1 coefficient vector, $\Sigma_{k}$ is the 4 × 4 covariance matrix of the input image, and **U** is a 4 × 4 identity matrix. $\mu_{k}$ is the mean of the input image and ${\bar{\hat{d}}}_{X}^{VIS}(k)$ is the mean of ${\hat{d}}_{X}^{VIS}(k)$. In case of Y and Z channels, the same method as the color-guided filtering of the X channel is applied.

**Figure 4:**
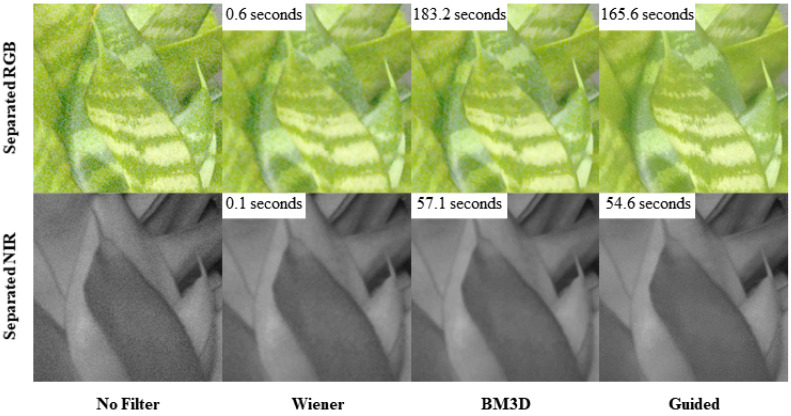
Subjective quality comparison of the denoising methods. The processing time is also shown.

Figure 4 shows the denoising results of the separated RGB and NIR images. Wiener [27] (Chapter 5) and BM3D [26] filters are applied to the separated images and the color-guided filter is applied by (17). Before the filtering process, the noise is seen obviously on the separated images. The Wiener filter reduces the noise, but also suffers from the effect of blurring along edges. In BM3D, the edge looks clearer than in the Wiener filter, but the texture on leaf in the separated RGB image looks blurrier than in the image without filtering. The image resulting from the guided filter looks better than the Wiener and BM3D filters by keeping the clarity of the edge and the texture on leaf.

## 3. Experimental Results

### 3.1. Experimental Condition

**Figure 5:**
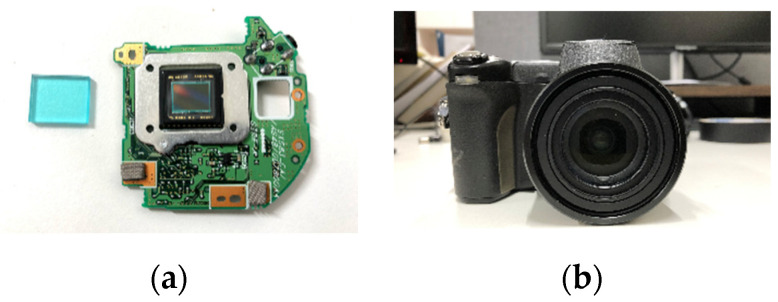
Imaging system used in the experiment. (**a**) The IR cut filter and imaging detector in the camera, (**b**) the camera used in the experiment.

To evaluate the experimental results of the proposed method, we used a CMYG CFA-based camera which is available on the consumer market (Figure 5b). The camera is modified by removing the IR cut filter from the camera. Figure 5a shows the hardware of the IR cut filter and the CMYG CFA-based detector inside of the camera. Test images were taken under three different light sources: fluorescent and 850 nm NIR light, a halogen light, and sunlight. Image processing software is implemented according to the proposed method. The color-guided filter-based [26] denoising method is applied to both separated XYZ and NIR images. The separated XYZ image is converted into the sRGB [31] space so that the resulting images are displayed in the sRGB color space. After all the processing, including CMYG to VIS/NIR separation, denoising, and color conversion, the output images are referred to as the separated RGB and the separated NIR images.

### 3.2. The Separation of Band Spectrum

**Figure 6:**
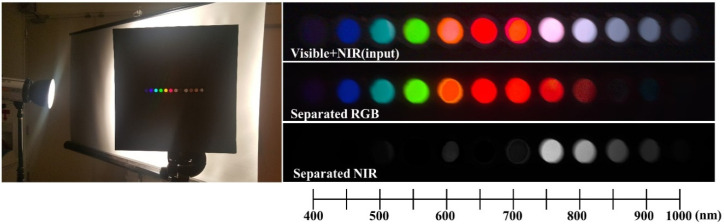
Experimental results of the separation of a band spectrum (bandwidth of the bandpass filters: 20 nm).

This experiment shows how well the visible and NIR images are separated from the mixed input image. Band pass filters from 400 to 1000 nm are aligned in a row and the camera captures the light passing through the filters from a halogen light source. Figure 6 shows the experimental environment and the separation results. From the result, the separated RGB image includes wavelengths from 400 to 800 nm. The separated NIR image includes data from 750 to 1000 nm. Due to degradation of spectral sensitivity of silicon-based sensors [35], the results in edge bands (400 nm and 1000 nm) look darker than the other bands. The result shows that the separated RGB and NIR images are overlapped from 750 to 800 nm, but the other bands are clearly separated. From this result, 750 nm is determined to be the starting wavelength of NIR, which is like several other conventional NIR imaging systems.

### 3.3. Separation under a NIR Spot Light

**Figure 7:**
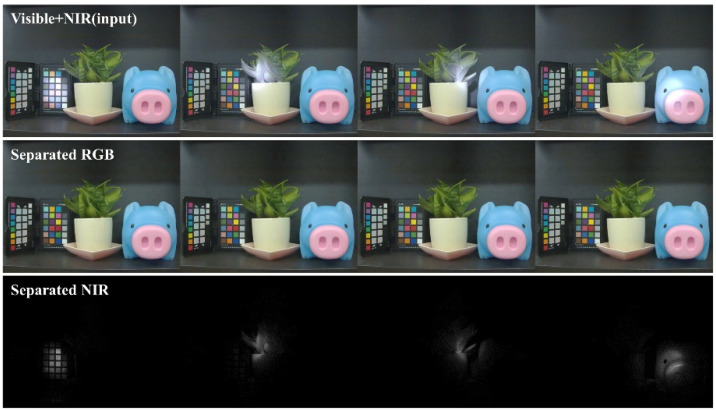
The separation experimental result under fluorescent and 850 nm NIR spot light illuminating four different locations from left to right.

To check the separation result under a combination of visible and NIR light sources, the images are captured under both fluorescent light and an 850 nm NIR flashlight. Figure 7 depicts the experimental result. The fluorescent light illuminates the entire area of the scene, but the NIR light shines only on a part of the scene to check the separation correctness of the NIR region. NIR light spots can be noted in the first row of Figure 7 from left to right. From the results, it is observed that the separated RGB images are similar because the fluorescent light illumination is identical in all images. On the other hand, the separated NIR images show different results, implying that the proposed method successfully separates the visible and NIR images even if the brightness between the visible and NIR light sources are changed.

### 3.4. Separation Results on Applications

**Figure 8:**
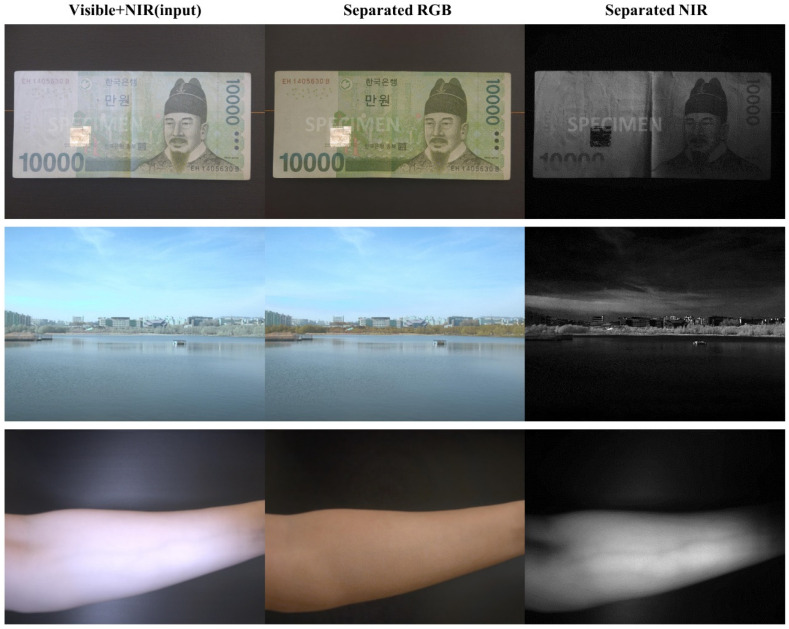
Examples of potential applications of the proposed method (Note: “SPECIMEN” text at banknote is overlapped on the images).

To evaluate the characteristics of the separated visible and NIR images, images were taken under several light sources. From the separation results, we can check how the separated images represent the characteristics of each spectrum band from the subjects. Figure 8 depicts the separation results from three different scenes. The separated NIR images are indicative of the special characteristics of NIR light.

Counterfeit money detection: The first row in Figure 8 shows the separation results on the image of a Korean banknote. The separated NIR image contains only texture information from certain parts of the bill. This NIR characteristic has been used to detect counterfeit banknotes [36].

Different NIR reflectance of subjects: The second row in Figure 8 was taken outside under sunlight. The scene consists of three different parts: the sky at the top, the building and trees in the middle, and the lake at the bottom. From the separated NIR image, the sky and the lake look relatively darker than the tree objects. According to the Rayleigh scattering [37], the wavelength of NIR is longer than the visible light, so the sky looks dark in the NIR image. At the bottom side, the lake looks dark in the separated NIR image because the water absorption of NIR light is higher than that of visible light [38,39]. On the other hand, the trees look brighter because the spectral reflectance of the NIR is higher than the visible light from the leaves of trees [40]. This characteristic has been applied to remote sensing applications for vegetation searches in a region [4].

Medical application: As one examples of medical applications, NIR images can be utilized to detect veins. The separated RGB image in the third row of Figure 8 shows a picture of an arm in which it is hard to detect the veins. On the other hand, the separated NIR image clearly indicates the shape of veins in the arm. NIR light reflection from the vein inside skin depends on the light intensity and thickness of tissue [41]. This application has been used for non-invasive medical diagnosis [9,10,42].

### 3.5. Objective Quality Comparison

**Figure 9:**
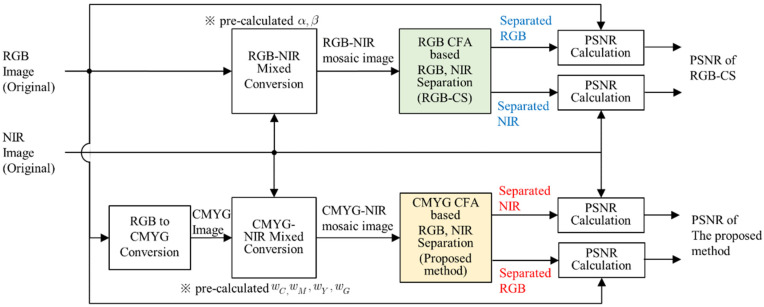
Block diagram of simulation method for the measurement of objective quality.

In this paper, objective quality performance of the proposed separation method is compared with a representative VIS-NIR separation method which is based on compressive sensing (RGB-CS) [21]. The imaging system used in this paper is CMYG CFA-based; however, the work for comparison [21] was applied to an RGB CFA-based imaging system. To minimize the differences in the experimental conditions due to the hardware difference of the imaging systems, a simulation is established to compare the objective performance between the two different approaches. Figure 9 shows the simulation structure for the objective quality measure.

To minimize differences between the method proposed in this work and the method used for comparison, mixed input images are generated from a pre-captured RGB and NIR image dataset [43]. The dataset was captured by a conventional digital camera with and without its IR cut filter removed. The original RGB images were captured with an IR cut off filter and the original NIR images were captured with IR long pass filter (and its IR cutoff filter removed). The input images were generated in two different ways. In the case of the proposed method, the color space of the input RGB image is converted to four channel CMYG color space. The converted CMYG image is then added to the NIR image to obtain the mixed input image. The weighting coefficients are pre-calculated, and the values are applied in the separation process. The mixed input image is converted to the mosaiced input image and the mosaiced input image is used as the input of the separation process. In case of the method used for comparison, the RGB and NIR inputs are added together using the mathematical representation of compressive sensing (RGB-CS) [21] and the pre-calculated weighting coefficients are applied in the same manner as the proposed method. In this paper, we implemented the separation process of RGB-CS by noting the description in the paper [21]. The performance of separated RGB and NIR images are measured with the input RGB and NIR images in terms of the peak-signal-to-noise-ratio (PSNR) [44] and color distance.

**Figure 10:**
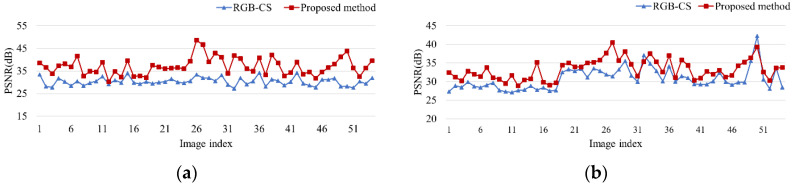
Peak-signal-to-noise-ratio (PSNR) comparison of RGB-CS [21] and the proposed method (**a**) The separated RGB image, (**b**) the separated NIR image.

PSNR comparison: Table 1 shows the simulation condition. In this paper, 54 sample RGB and NIR images are used for the simulation. Figure 10 depicts the PSNR comparison graphs between the two methods. From the simulation results, the PSNR of the proposed method is higher than that of RGB-CS. The average PSNR of the separated RGB and NIR images of the proposed method is 37.04 dB and 33.29 dB, respectively. On the other hand, the average PSNR of the separated RGB and NIR images of RGB-CS is 30.32 dB and 30.74 dB, respectively. According to the average PSNR comparison, the objective quality of the proposed method is 6.72 and 2.55 dB higher, respectively, than RGB-CS.

Color distance comparison: To compare color differences between two images, color distance between two colors in sRGB, XYZ, and CIELab [46] color spaces are calculated. In this paper, the color distance is calculated by following average of Euclidean distance:

(20) $$\begin{array}{l} CD_{RGB}=\frac{1}{N}\sum_{i=1}^{N}\sqrt{{\left(R_{i}^{org.}-R_{i}^{sep.}\right)}^{2}+{\left(G_{i}^{org.}-G_{i}^{sep.}\right)}^{2}+{\left(B_{i}^{org.}-B_{i}^{sep}\right)}^{2}}\\ CD_{XYZ}=\frac{1}{N}\sum_{i=1}^{N}\sqrt{{\left(X_{i}^{org.}-X_{i}^{sep.}\right)}^{2}+{\left(Y_{i}^{org.}-Y_{i}^{sep.}\right)}^{2}+{\left(Z_{i}^{org.}-Z_{i}^{sep}\right)}^{2}}\\ CD_{CIELab}=\frac{1}{N}\sum_{i=1}^{N}\sqrt{{\left(L_{i}^{org.}-L_{i}^{sep.}\right)}^{2}+{\left(a_{i}^{org.}-a_{i}^{sep.}\right)}^{2}+{\left(b_{i}^{org.}-b_{i}^{sep}\right)}^{2}}\\ CD_{RGB}^{histogram}=\frac{1}{M}\sum_{i=1}^{M}\sqrt{{\left(Rhist._{i}^{org.}-Rhist._{i}^{sep.}\right)}^{2}+{\left(Ghist._{i}^{org.}-Ghist._{i}^{sep.}\right)}^{2}+{\left(Bhist._{i}^{org.}-Bhist._{i}^{sep}\right)}^{2}}\\ CD_{XYZ}^{histogram}=\frac{1}{M}\sum_{i=1}^{M}\sqrt{{\left(Xhist._{i}^{org.}-Xhist._{i}^{sep.}\right)}^{2}+{\left(Yhist._{i}^{org.}-Yhist._{i}^{sep.}\right)}^{2}+{\left(Zhist._{i}^{org.}-Zhist._{i}^{sep}\right)}^{2}}\\ CD_{CIELab}^{histogram}=\frac{1}{M}\sum_{i=1}^{M}\sqrt{{\left(Lhist._{i}^{org.}-Lhist._{i}^{sep.}\right)}^{2}+{\left(ahist._{i}^{org.}-ahist._{i}^{sep.}\right)}^{2}+{\left(bhist._{i}^{org.}-bhist._{i}^{sep}\right)}^{2}} \end{array}$$

where $CD_{RGB}$, $CD_{XYZ}$, and $CD_{CIELab}$ are the color distance in each sRGB, XYZ, and CIELab color space, respectively. $CD_{RGB}^{histogram}$, $CD_{XYZ}^{histogram}$, and $CD_{CIELab}^{histogram}$ are the color distance of histogram in each color space. *N* is the number of pixels in image and *M* is the number of bins in the histogram. The bin size of histogram is 256 in this paper. In case of NIR image, the intensity distance is also calculated by

(21) $$\begin{array}{l} ID_{NIR}=\frac{1}{N}\sum_{i=1}^{N}\left(NIR_{i}^{org.}-NIR_{i}^{sep.}\right)\\ ID_{NIR}^{histogram}=\frac{1}{M}\sum_{i=1}^{M}\left(NIRhist._{i}^{org.}-NIRhist._{i}^{sep.}\right) \end{array}$$

where $ID_{NIR}$ and $ID_{NIR}^{histogram}$ are the intensity distance of NIR images and its histogram, respectively.

**Figure 11:**
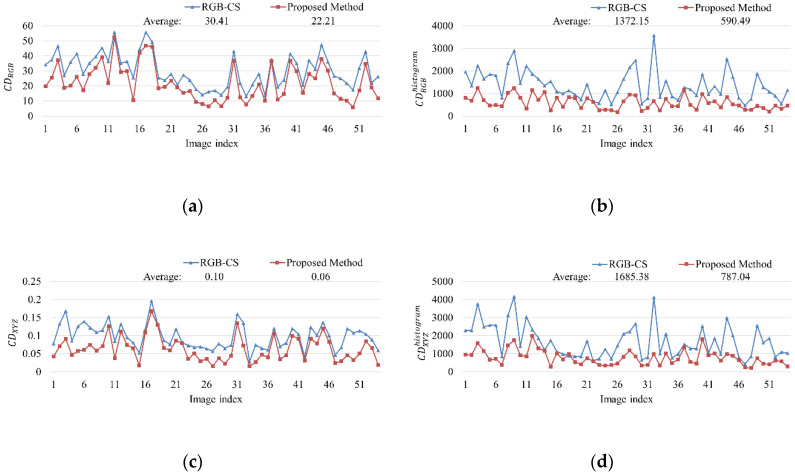

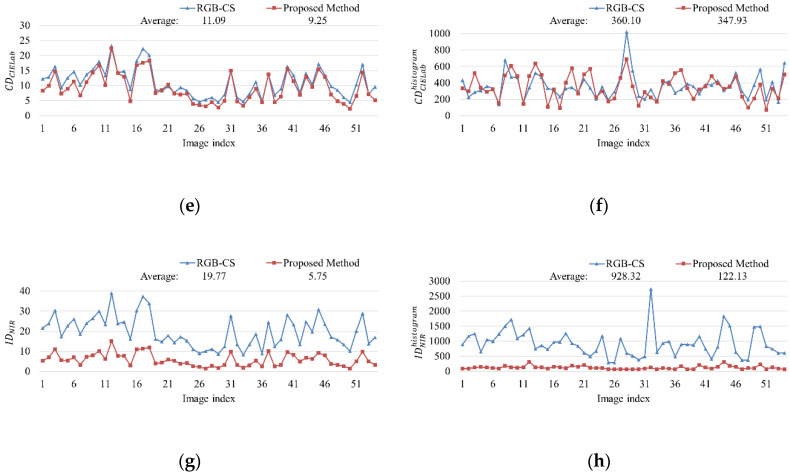
Color distance comparison of RGB-CS [21] and the proposed method (**a**) sRGB space, (**b**) Histogram in sRGB space, (**c**) XYZ space, (**d**) Histogram in XYZ space, (**e**) CIELab space, (**f**) Histogram in CIELab space, (**g**) intensity distance of NIR image, (**h**) Histogram distance of NIR intensity.

Figure 11 depicts results of color distance comparison between RGB-CS and the proposed method. The results show that the proposed method has lower color distance value than RGB-CS method. This means that the color difference between original and separated images of the proposed method looks more similar than RGB-CS method.

**Figure 12:**
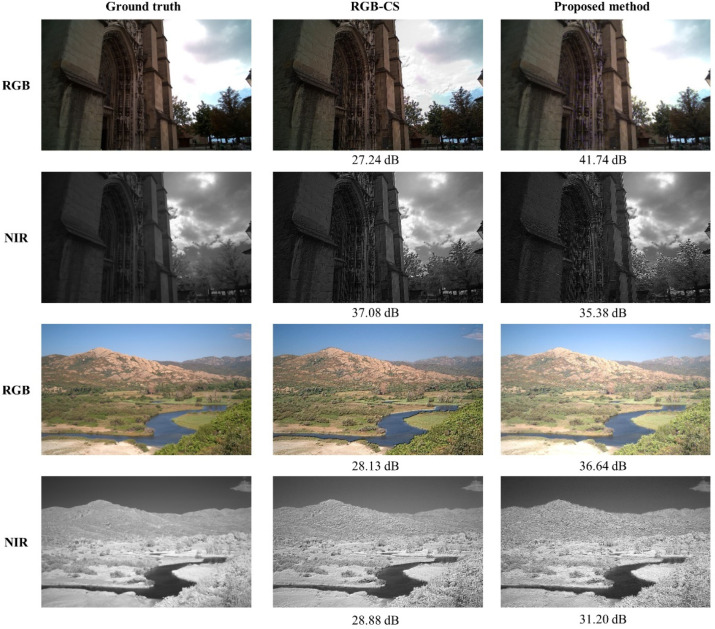
The subjective quality comparison between RGB-CS [21] and the proposed method (Note: the result images were encoded by portable network graphics (PNG) format).

Figure 12 depicts the subjective quality comparisons between RGB-CS and the proposed method. In Figure 12, both methods show separated RGB and NIR images pretty well, but in case of RGB, more differences were found between RGB-CS and the ground truth images. The RGB results by RGB-CS looks sharper than the proposed method, but careful comparison with the ground truth reveals that the high frequencies of RGB-CS are over-emphasized (see the cloud in the images in the first row, for example). It means that the proposed method generates separated results more faithful to the ground truth.

## 4. Conclusions

In this paper, visible and NIR image separation from a single image is proposed. The image is captured with a CMYG CFA-based camera that is modified by removing the IR cut filter in front of the detector. The proposed method is performed in a simple way by multiplying a separation matrix with the input pixels. After the separation, the separated XYZ can be converted into a chosen color space for display, in this work, the sRGB color space is used. To reduce the noise after the separation process, we analyzed the noise characteristics in the separated images and a color-guided filter [26] was applied for denoising using the CMYG input as the guided image. The experimental results by testing with several band pass filters and light sources show that the visible and NIR images are successfully separated. The use of the proposed method is also implemented for three applications: counterfeit money, vegetation, and vein detection. To measure the objective quality in terms of PSNR, the separation performance of the proposed method and RGB-CS [21] is simulated. The simulation results show that the proposed method achieved 6.72 and 2.55 dB higher PSNR than RGB-CS on the separated RGB and NIR images, respectively.

Discussion and future work: In this paper, we proposed four channels of visible and NIR separation from CMYG CFA. However, there is a limitation to the dynamic range of sensor and bit-depth if the mixture of the color bands contains more channels than can be separated. A novel approach should be invented to overcome this limitation as future work. Nevertheless, the proposed separation method can be improved with better color filter array mixtures by increasing the number of equations. If the spectral bands need to be increased for a certain application, the proposed method might be extended to capture more color bands in a single sensor, which needs to be investigated as future work as well.

## Figures and Tables

**Table 1:** The Simulation Conditions.

Method	RGB-CS [21]	The Proposed Method
Sample image info.	54 images (508 × 768–1024 × 762) [43]	
Type of CFA ^1^	RGB	CMYG
Separation matrix	Pre-defined	
Sparsifying matrix	Discrete cosine transform	N/A
Demosaicing	Bilinear (GRBG pattern)	Bilinear (GMYC pattern)
Separation method	SL0 sparse decomposition [45]	Matrix multiplication

^1^ RGB means that the input image is generated by considering the RGB CFA-based system and CMYG is by considering the CMYG-CFA.
